# The biological function of type I receptors of bone morphogenetic protein in bone

**DOI:** 10.1038/boneres.2016.5

**Published:** 2016-04-05

**Authors:** Shuxian Lin, Kathy K H Svoboda, Jian Q Feng, Xinquan Jiang

**Affiliations:** 1 Department of Prosthodontics, Ninth People’s Hospital Affiliated with Shanghai Jiao Tong University, School of Medicine, Shanghai, China; 2 Department of Biomedical Sciences, Texas A&M Baylor College of Dentistry, Dallas, TX, USA

## Abstract

Bone morphogenetic proteins (BMPs) have multiple roles in skeletal development, homeostasis and regeneration. BMPs signal via type I and type II serine/threonine kinase receptors (BMPRI and BMPRII). In recent decades, genetic studies in humans and mice have demonstrated that perturbations in BMP signaling via BMPRI resulted in various diseases in bone, cartilage, and muscles. In this review, we focus on all three types of BMPRI, which consist of activin-like kinase 2 (ALK2, also called type IA activin receptor), activin-like kinase 3 (ALK3, also called BMPRIA), and activin-like kinase 6 (ALK6, also called BMPRIB). The research areas covered include the current progress regarding the roles of these receptors during myogenesis, chondrogenesis, and osteogenesis. Understanding the physiological and pathological functions of these receptors at the cellular and molecular levels will advance drug development and tissue regeneration for treating musculoskeletal diseases and bone defects in the future.

## Introduction

Belonging to the transforming growth factor-β super family,^1^ bone morphogenetic proteins (BMPs) were discovered and named in 1965 by Marshall Urist, who initially identified their ability to induce ectopic bones in muscles.^2^ In the last 50 years, the potent osteogenic activity *in vitro* of BMPs has been well characterized,^3^ as well as their constitutive activation or exogenous application, which can induce ectopic bone formation *in vivo*.^4,5^ BMPs signal through cell-surface receptor complexes that consist of two distinct transmembrane serine/threonine kinase receptors, type I (BMPRI) and type II (BMPRII).^6^ Initially, BMP ligands bind with high affinity to BMPRI, followed by heterodimerization with BMPRII, which allows the BMPRII to phosphorylate a short stretch of amino acids in the BMPRI and activate kinase activity.^6^ Classically, after the activation of BMPRI, intracellular signaling is initiated through the phosphorylation of the C-terminal SSXS motif of specific receptor-regulated Smads, including Smad1, 5, and 8.^7–10^ After being released from the receptor, the phosphorylated Smads form heteromeric complexes with common partner Smad, that is, Smad4. This complex is then translocated into the nucleus to regulate the transcription of genes, broadly influencing growth and differentiation.^9^

**Figure 1:**
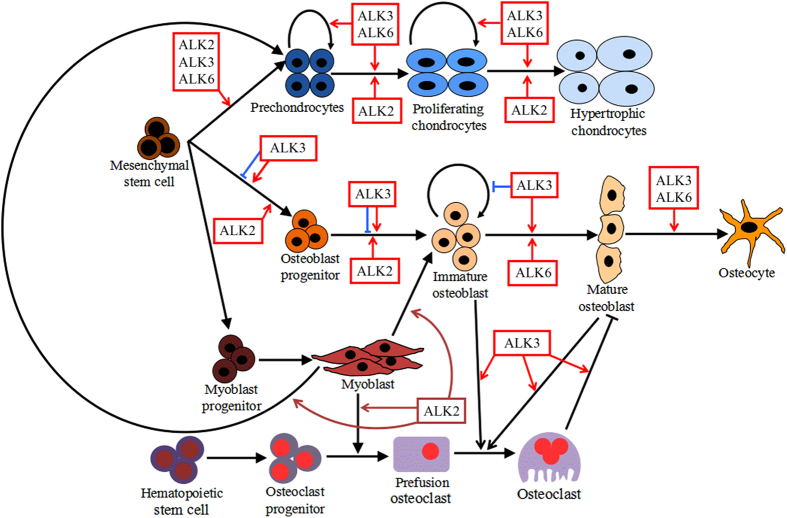
Regulatory roles of ALK2, ALK3, and ALK6 in the various differentiation stages of osteolineage, chondrolineage, and osteoclast lineage cells and myoblasts. Osteoblasts, chondrocytes, and myoblasts are derived from mesenchymal progenitor cells, whereas osteoclasts are derived from hematopoietic precursors. BMP, bone morphogenetic protein.

Three type I receptors have been shown to effectively bind BMP ligands during mammalian skeletal development—types IA and IB BMP receptors (BMPRIA or ALK3 and BMPRIB or ALK6), as well as type IA activin receptor (ACVRI or ALK2).^11,12^ In recent decades, studies of clinical patients, genetic animal models and cell lines have consistently demonstrated that all three type I receptors are essential for osteolineage and chondrolineage proliferation, differentiation and function. It has become clear that alterations in the intensity, location, and duration of BMPRI activity lead to heterotopic bone formation, skeleton, and cartilage deformation, as well as bone metabolism disorders. Here we provide an updated review that specifically focuses on the biological function of BMPRI in bone formation. Emphasis is placed on murine genetic studies (Table 1) that have assessed the requirement for and the roles of different types of BMPRI, including ALK2, ALK3, and ALK6 during osteogenesis, chondrogenesis and osteoclastogenesis, as summarized in Figure 1.

## Biological functions of ALK2 in bone formation

ALK2 is widely expressed in many tissues during embryonic development and is highly present in bones during postnatal development.^13^ Several mesenchymal stem cell (MSC) lines show high expression levels of ALK2,^11,14^ and its constitutive activation in myoblasts induces heterotopic bone during the endochondral bone formation process, suggesting that ALK2 has essential roles in both osteogenesis and chondrogenesis.

### Ectopic expression of ALK2 in myoblasts leads to heterotopic endochondral bone formation

The regulatory role of ALK2 in osteogenesis and chondrogenesis did not arouse interest until the discovery of fibrodysplasia ossificans progressiva (FOP, MIM 135100), which is characterized by congenital malformations of the great toes and progressive heterotopic ossification in muscles, tendons, ligaments, and other connective tissues.^15,16^ Genetic analysis of FOP patients has identified gain-of-function mutations in *ALK2*, including c.617G>A (p.R206H), c.619C>G (p.Q207E), c.1067G>A (p.G356D), c.982G> T(p.G328W), c.983G> A(p.G328E), c.982G>A (p.G328R), c.774G>C/c.774G>T (P.R258S), c.1124G>C (p.R375P), c.587T>C (p.L196P), c.590–592delCTT (p.P197_F198delinsL), and c.605G>T (p.R202I), among which R206H is the most common mutation and can be found in ~90% of FOP patients.^17–31^ The classic, constitutively active ALK2 receptor containing the artificial Q207D mutation or the R206H mutation recaptures the FOP condition in transgenic animal models.^32,33^ Further evaluation of these FOP mutations revealed that the ectopic expression of ALK2 increased Smad-dependent BMP signaling activity, which potentially occurs for both osteogenic and chondrogenic differentiation of myoblasts, thus forming heterotopic bone through an endochondral bone formation process.^21,32,34–39^ In addition, inhibiting the activation of BMP signaling effectors Smad1/5/8 in tissues that constitutively express ALK2 resulted in a reduction of the ectopic ossification and functional impairment.^32^ The stable *in vitro* transfection of the *Alk2*^*R206H*^ mutation in C2C12 cells (mouse myoblasts) increased the levels of both osteogenic markers (*osterix* (*Osx*), *alkaline phosphatase* (*Alp*)) and chondrogenic markers (*type II collagen* (*Col2*), *type X collagen* (*Col10*)).^40,41^ Conversely, knockdown of *Alk2* in C2C12 cells potentiated muscle differentiation and repressed BMP6-induced osteoblast differentiation.^42^ These elevated results suggest that Smad-dependent ALK2 signaling is important in heterotopic ossification and endochondral bone formation of myoblasts.

### ALK2 regulates osteogenic and chondrogenic differentiation of MSCs

Studies focusing on *Alk2*^*R206H*^ mutant mice or cells also provide evidence indicating that ALK2 has an important role in the osteogenic differentiation of MSCs. First, the mesenchymal progenitor cells isolated from FOP (R206H) patients or *Alk2*^*R206H*^ mutant mice showed increased Smad-dependent BMP signaling activity with upregulated *Alp*, *runt-related gene 2* (*Runx2*), and *osteocalcin* (*Ocn*) genes.^39,43^ Second, the constitutive expression of *Alk2* in mesenchymal cells or pre-osteoblasts makes those cells more receptive to exogenous BMPs with respect to differentiating into functional mineralizing osteoblasts.^41^ In contrast, specifically suppressing *Alk2* activity decreased the enhanced osteogenic differentiation to control levels.^43^ Furthermore, *in vivo* studies found that in heterozygous *Alk2*^*R206H*^ knock-in mice, the Tek/Tie2+ progenitor cells could be recruited and differentiated into bone cells in heterotopic ossification lesions.^33^ Consistently, a recent mouse model study showed that conditional activation of *Alk2* in mesodermal lineage cells resulted in ectopic bone formation at distal joints with an elevated number of osteoblast progenitors as well as bone formation activity.^44^ Collectively, Smad-dependent ALK2 signaling in the mesenchymal progenitors has an important role for their specification toward osteolineage cells.

In addition, the tracking study of Tek/Tie2+ progenitor cells in heterozygous *Alk2*^*R206H*^ knock-in mice also identified a chondrogenic differentiation of this cell population, which was responsible for forming a cartilage template that developed to form endochondral bone.^33^ Further analysis found that ectopic expression of *Alk2* increased sensitivity and accelerated chondrogenic differentiation of mouse embryonic fibroblasts and that the loss of *Alk2* severely inhibited chondrogenic differentiation, suggesting that ALK2 was required during early chondrogenesis.^39^ Moreover, studying a chick limb bud with constitutive *Alk2* expression showed accelerated chondrocyte maturation and induced Indian hedgehog, which is a key factor for chondrocyte maturation.^45^ Moreover, chondrocytes with the *Alk2*^*R206H*^ mutation showed increased expression of both the early chondrocyte-specific markers *(sex determining region Y)-box 9* (*Sox9*), *Col2*, *aggrecan* (*Agg*), and the late marker *Col10*.^39^ Collectively, these results established that ALK2 is an essential enhancer of chondrogenic differentiation.

### Roles of ALK2 in regulating osteoblasts and osteoclasts

**Figure 2:**
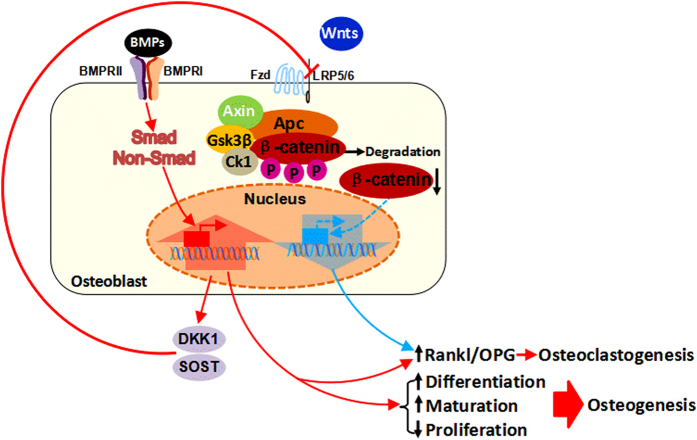
A proposed mechanism diagram describing the crosstalk between osteoblastic BMP signaling (mainly via ALK3) and canonical Wnt signaling in regulating bone homeostasis. After being activated by BMPs, the BMPRs in the cell surface induce intracellular BMP signaling, including both Smad-dependent signaling and non-Smad-dependent signaling. Then, these activated signaling pathways initiate the expression of canonical Wnt inhibitors (DKK1 and SOST), which influence the binding of Wnt ligands to their receptor complexes consisting of low-density lipoprotein (LDL) receptor-related protein 5/6 (LRP5/6) and frizzled (Fzd) receptors. As a result, cytoplasmic β-catenin will be degraded, and its transcriptional regulation will be diminished, resulting in a downregulation of Wnt/β-catenin signaling activity. The balance between BMP and canonical Wnt signaling affects bone development and homeostasis by regulating both osteogenesis and osteoclastogenesis.

The endogenous expression level of *Alk2* in postnatal bone was found to be much higher than that in heart and skeletal muscles,^13^ suggesting that ALK2 might also be essential in osteolineage cells. Accordingly, *Alk2* knockdown in murine osteoblast progenitors (KS483) reduced BMP6-induced osteogenic differentiation.^42^ Interestingly, an *in vivo* study found that a conditional disruption of *Alk2* in bone cells (including immature osteoblasts, mature osteoblasts, and osteocytes) led to an increase in endogenous bone mass during postnatal development.^13^ Analysis of this mouse model indicated that the disturbed bone homeostasis was more likely due to an upregulation of canonical Wnt signaling in conjunction with the downregulation of Wnt inhibitors, scelerostin (SOST) and dickkopf 1 (DKK1; Figure 2).^13^ In addition to BMP signaling, Wnt signaling in osteoblasts has been examined for a decade, and there is sufficient evidence supporting the hypothesis that canonical Wnt signaling serves as a bone mass inducer that positively regulates osteoblast differentiation and maturation but negatively affects osteoclast activity (see below for a detailed description).^46^ However, the direct regulatory role of ALK2-induced BMP signaling in late osteolineage cells remains unclear, and further evaluations need to be performed for a comprehensive understanding.

To date, no evidence has demonstrated that ALK2 directly regulates osteoclast function. However, recent studies have found that the constitutively active mutation of ALK2 in myoblasts led to the increased formation of osteoclasts from their precursors through transforming growth factor-β signaling. An implantation of *Alk2*^*R206H*^-transfected C2C12 cells with BMP2 in nude mice resulted in robust heterotopic ossification with increased osteoclast formation in muscle tissues.^47,48^ Furthermore, a co-culture of *Alk2*^*R206H*^-transfected C2C12 cells as well as the conditioned medium from *Alk2*^*R206H*^-transfected C2C12 cells enhanced osteoclast formation in mouse monocytic RAW264.7 cells.^47^ Mechanism analysis suggested that the elevated secretion of transforming growth factor-β from the mutant myoblasts led to the upregulated activation of p38 mitogen-activated protein kinase (MAPK) signaling in the surrounding monocytes, thus contributing to the enhanced osteoclastogenic differentiation.^47^

## Biological functions of ALK3 in bone formation

ALK3 is widely expressed in a variety of tissues during embryonic development, but it is mainly expressed in osteolineage cells and bone marrow cells during postnatal bone formation, based on *in vitro* studies of different cell lines.^49,50^ Findings have confirmed a high expression level of ALK3 in osteolineage cells from MSCs to differentiated bone cells.^14,51–54^ Many studies have consistently indicated that ALK3 is one of the key receptors for conducting BMP signaling during osteogenesis and chondrogenesis. However, the roles of ALK3 in bone biology have remained unclear until recent studies using *Alk3* conditional ablation in osteogenic tissues because its conventional deletion in mice is embryonically lethal before bone development.^55^ It is clear that the regulatory role of ALK3 differs depending on distinctive cells, stages, tissues, and ages. ALK3-induced BMP signaling also crosstalks with the Wnt/β-catenin signaling pathway and functions in interactions between osteoblasts and osteoclasts.

### Roles of ALK3 in mesenchymal pre-osteolineage cells

In MSCs, the forced expression of *Alk3* (that is, the overexpression of wild-type *Alk3*) initiated osteogenic development. On the contrary, downregulating *Alk3* activity (that is, overexpressing truncated *Alk3*) led to decreased expression of *Alp* and less von Kossa staining.^51^ When specifically deleting *Alk3* in the early development of the palatal mesenchyme by E12.5 using *Oar2-Ires Cre*, the formation of mesenchymal condensation in the palate was delayed as was the consequent palate bone formation.^56^ This *in vivo* study, together with the *in vitro* study, suggested that ALK3-induced BMP signaling was required for the differentiation of MSCs toward the osteolineage. However, other groups came to an opposite conclusion in similar studies. The downregulation of *Alk3* in 2T3 cells (characterized as osteoblast precursors) or the conditional deletion of *Alk3* in bone marrow mesenchymal cells using *Mx1 Cre* (Cre is activated in an osteolineage-restricted stem/progenitor cell subset, one specific subset of bone marrow mesenchymal cells^57^) led to ectopic mineralization via the upregulation of the bone formation activity of osteoblasts,^49,54^ indicating that ALK3 inhibits the osteoblastic lineage commitment of bone marrow stem cells. These contrary results suggest that ALK3 may regulate (promote or inhibit) the differentiation of MSCs in a tissue-dependent manner.

### Roles of ALK3 in osteoblasts

Deletion of *Alk3* specifically in immature osteoblasts using *2.3 kb Col1 Cre* or *3.2 kb Col1 Cre* indicated that the maturation progress of these osteoblasts was suppressed because both the bone formation rate and the mineral apposition rate were downregulated.^58–60^ An analysis of these mutant osteoblasts found an enhanced proliferation with decreased expression of several specific osteoblast markers, including *Runx2, Osx, bone sialoprotein* (*Bsp*), and *Alp*.^58–60^ Mice with a specific disruption of *Alk3* in differentiated osteoblasts (using *Og2 Cre*) were born normally and did not exhibit overt bone changes, except for a slight decrease in bone mass at 3 months, which may be caused by a mild downregulation of osteoblast function (lower bone formation rate with decreased BV/TV, but no change in the expression levels of *osteopontin* (*Opn*) and *Ocn*),^61^ suggesting that ALK3 promotes mature osteoblast function and osteoblast–osteocyte transition in a relatively mild way. However, the decreased bone mass in these mutant mice later increased and was confirmed to have an even higher bone volume compared with that of wild-type mice at 10 months, which mainly resulted from the downregulated bone resorption caused by reduced osteoclast activity.^61^ These results suggest that ALK3 expressed in mature osteoblasts has diverse effects on bone mass and homeostasis in an age-dependent manner.

### Roles of ALK3 in the interaction between osteoblasts and osteoclasts

For decades, more and more studies have confirmed that there is a communication between osteoblasts and osteoclasts, which has an exquisite and important role in bone modeling and remodeling. The discoveries of the biological functions of ALK3 imply that this factor may be one of the key molecules in this process.

Osteoblasts have critical roles in bone resorption by regulating osteoclastogenesis due to their ability to produce nuclear factor kappa-B ligand (RANKL), which is essential for promoting osteoclast differentiation and function, and its decoy receptor osteoprotegerin (OPG).^62,63^ Several studies have confirmed that ALK3-induced signaling in osteoblasts regulates osteoclastogenesis via the RANKL-OPG mechanism. The earliest direct evidence came from a report by Wan *et al.*,^64^ in which they found that the transfection of constitutively active *Alk3* stimulated the OPG promoter and that two homeobox C8 (Hoxc-8)-binding sites in the OPG promoter responded to the ALK3 activation. In accordance with these results, several *in vivo* studies have confirmed that conditional deletion of osteoblastic *Alk3* in distinct cell differentiation stages,^59,60,61^ or in different developmental periods,^58,60,61,65^ led to decreased osteoclast numbers and decreased expression of bone resorption markers (*matrix metallopeptidase 9* (*Mmp9*), *tartrate-resistant acid phosphatase* (*Trap*), and *cathepsin K* (*CatK*), among others). Furthermore, accumulating evidence suggests that crosstalk between BMP and Wnt signaling in bone may also be involved in osteoblast-regulated osteoclastogenesis through the RANKL-OPG pathway. Kamiya *et al.*^60^^,^^65^ recognized that a disruption of ALK3-induced signaling, including both Smad and non-Smad signaling (such as p38 MAPK), in osteoblasts resulted in upregulated Wnt/β-catenin activity due to decreased production of its downstream targets, DKK1 and SOST.^60,65^ It is widely reported that canonical Wnt signaling in osteoblasts negatively regulates their supporting function in osteoclastogenesis by affecting RANKL and OPG expression, thus inhibiting osteoclast differentiation and activity as well as suppressing osteoclast-mediated bone resorption.^66,67^ Taken together, in osteoblasts, ALK3 activates SOST and DKK1, while both SOST and DKK1 inhibit canonical Wnt signaling and maintain the activity of Wnt/β-catenin signaling at a certain level.^68–71^ As a result, ALK3-induced BMP signaling and Wnt signaling contribute not only to osteoblast proliferation, differentiation, and maturation, but also to the regulation of osteoclastogenesis (mainly via the RANKL-OPG pathway; Figure 2).

However, ALK3 signaling in osteoclasts also negatively regulates osteoblast functions. For example, conditionally disturbed *Alk3* expression in osteoclasts using *CatK Cre* not only inhibited osteoclast function but also enhanced osteoblast function, that is, increased osteoblast number and bone formation rate.^59^ Furthermore, it has been reported that several factors (including platelet-derived growth factor BB, v-ATPase V0 subunit d2 (Atp6v0d2), CatK and osteoclast inhibitory lectin (OCIL)) produced by osteoclasts negatively affect osteoblast functions.^72–75^ Among these osteoclast-derived osteoblastic inhibitors, both CatK and ATV6v0d2 were significantly increased after a stimulation of Smad-dependent ALK3 signaling in osteoclasts.^76–78^ Collectively, BMPs might bind to ALK3 on the surface of osteoclasts and activate BMP signaling, leading to the upregulation of factors such as CatK and ATV6v0d2, which suppress osteoblast activity and downregulate the rate of bone formation.^76,77,79^

### Roles of ALK3 in chondrogenesis

Although ALK3 mainly functions in osteogenesis, it also has an important role in chondrogenesis. For instance, a forced expression of *Alk3* in MSCs (C3H10T1/2)^51^ or pre-chondrocytes^80^ induced chondrogenic differentiation, while downregulated *Alk3* suppressed this process.^51^ The regulatory function of ALK3 in chondrogenesis has been further supported by *in vivo* studies.^81–86^ Initially, the role of ALK3 in chondrogenesis, such as regulating chondrocyte proliferation, survival, and differentiation, was thought to be associated with ALK6 during chondrogenesis.^81^ Soon after, it was demonstrated that ALK3 itself has a unique and broad role during chondrogenesis. First, overexpressing a constitutively active ALK3 in chondrocytes *in vivo* stimulated the differentiation of pre-chondrocytes and proliferating chondrocytes, promoting their maturation toward hypertrophy.^82^ Second, the conditional deletion of *Alk3* specifically in developing joints resulted in the downregulation of proteoglycans and extracellular matrix cartilage genes, including *Col2*, *Col10*, and *Agg*, leading to articular cartilage fibrosis and degeneration during postnatal development.^83^ Third, *Alk3* ablation in postnatal chondrocytes caused arrested chondrogenesis and endochondral ossification, with diminished chondrocyte proliferation and little expression of cartilage markers, such as SOX9, Indian hedgehog, Col II, Col X, AGG, and glycoproteins, among others.^84–86^ Taken together, these studies support the notion that ALK3 is one of the key factors for regulating the specification of pre-chondrogenic mesenchyme as well as chondrolineage differentiation and maturation, postnatal chondrogenesis and the maintenance of articular cartilage.

## Biological functions of ALK6 in bone formation

The expression of ALK6 is primarily restricted to mesenchymal pre-cartilage condensations during mouse development, but it has also been identified in differentiated chondrocytes and osteoblasts in adult mice.^50,87–89^ Accordingly, a study of different cell lines suggested that *Alk6* was expressed at a low level or was undetectable in MSCs; however, it was specifically upregulated during osteoblastic differentiation.^14,51,53^ These ALK6 expression patterns suggest that it may participate in chondrogenesis and influence late osteolineage cells.

### ALK6 in regulating chondrogenesis

Patients with a homozygous mutation in *ALK6* have severe limb deformations consisting of a short stature, aplasia of the fibula, severe brachydactyly and ulnar deviation of the hands, which mainly result from chondrodysplasia during skeletal development.^90^ In addition, constitutive expression of *Alk6* in pre-chondrocytes significantly increases the induction of chondrocyte differentiation.^80,91^ Accordingly, several *in vivo* studies have consistently demonstrated that ALK6 is required for the proliferation and chondrogenic differentiation of pre-chondrogenic and proliferating chondrocytes.^82,88^ However, null mutations of *Alk6* only exhibited mild limb abnormalities that were largely restricted to the appendicular skeleton.^82,88,92^ Soyun Yi^88^ posited that ALK6 had broadly overlapping functions with other BMP receptors because a *Alk6*-*Bmp7* double-mutant exhibited more-severe skeletal defects than did an *Alk6* single-knockout. Later, it was suggested that ALK3 and ALK6 display functionally redundant aspects during early chondrogenesis^81^ because ALK6 signaling could be replaced by constitutively active ALK3.^82^ In summary, these results indicate that ALK6, rather than having a unique role, may have overlapping functions with other BMP receptors, especially ALK3, in supporting pre-chondrogenic mesenchyme as well as chondrocyte proliferation, differentiation, and maturation.

### ALK6 in regulating osteogenesis

*In vitro* studies have found that the expression of a constitutively active *Alk6* induced the formation of mineralized bone matrix, while the overexpression of truncated *Alk6* or the inhibition of endogenous *Alk6* completely blocked BMP2-induced osteoblast differentiation and mineralized bone matrix formation.^54^ These results suggest that the osteoblastic ALK6 is required for osteoblast differentiation and bone formation. Transgenic mice that expressed a truncated dominant-negative *Alk6* in targeted osteoblasts using the *2.3 kb Col1* promoter exhibited impaired postnatal bone formation, including severely reduced bone mineral density, bone volume, and bone formation rates. These characteristics indicate that the osteoblastic ALK6 has a necessary role during postnatal bone modeling and remodeling via regulating osteoblast/osteocyte maturation.^93^ However, in *Alk6* null mice, the defects largely resulted from the disturbed chondrogenesis, and there was little influence on osteogenesis,^88^ implying that the regulatory role of ALK6 is mildly involved in bone ossification.

## Conclusion

In conclusion, all three types of BMPRI have distinct but important roles during chondrogenesis, osteogenesis, and osteoclastogenesis. They might not only directly regulate the chondrogenic or osteogenic differentiation of bone cells and influence osteoclast activity through the RANKL-OPG pathway but also crosstalk with Wnt signaling by altering their downstream molecules, including DKK1 and SOST, during bone development and homeostasis. Despite some knowledge gaps, much has been learned over recent decades about the functions of BMPRI in a variety of cell types, including MSCs, chondrocytes, osteoblasts, osteoclasts, and myoblasts, using genetic animal models. However, its regulatory role in osteocytes remains unknown. Although osteocytes, which compose 90%–95% of all bone cells in adult bone, have recently been demonstrated to be crucial for bone biology because of their functions in inducing osteoclasts, regulating mineral metabolism and matrix remodeling, and reacting to mechanical loading.^94^ Furthermore, studies of BMPRI have been fueled by the desire to understand the molecular underpinnings of rare bone diseases or the mechanisms of clinical applications for BMPs in common diseases, such as bone fracture healing and spinal surgery, and these studies now might contribute to the development of new therapies for congenital or age-related bone diseases. Recently, Marc Baud’huin *et al.*^95^ developed a soluble mBMPRIA (ALK3)-mFc fusion protein and found that mBMPRIA (ALK3)-mFc treatment could successfully downregulate Smad-dependent ALK3 signaling, thus increasing bone mass in both young (7–10 weeks) and old (14–18 weeks) mice or preventing bone loss induced by estrogen deficiency in ovariectomized mice. This work set an example showing that regulation of signaling through BMPRI may have therapeutic benefits. Hence, continuing the bedside-to-bench exchange of information about BMPRI will help to provide novel, therapeutically useful strategies for skeletal physiology, pathology, and regeneration.

## Figures and Tables

**Table 1:** Summary of skeletal phenotypes in mouse models with BMPRI alterations

Gene	Tg/KO/KI/CKO/CKI	Promoter/Cre line	Stage	BMP signal	Bone and cartilage phenotype(s)	References
*ALK2*	CKI (*Alk2*^*Q207D*^)	Ad.Cre (injection)	P7–P30	Up	Heterotopic endochondral ossification	^32^
	CKI (*Alk2*^*Q207D*^)	CAGGS Cre^ER^	P7–P60	Up	Heterotopic endochondral ossification	^32^
	Het KI (*Alk2*^*R206H*^)		6–8 w	Up	Heterotopic endochondral ossification	^33^
	CKO	3.2 kb Col1 Cre^ER^	E13.5–E18.5, P2–P21	Down	Bone mass ↑	^13^
	CKI (*Alk2*^*R206H*^)	Nfatc1 Cre	P4–P40	Up	Ectopic cartilage and bone at the distal joints	^44^
*ALK3*	KO		E7.0, E8.5	Down	Embryonic lethality	^55^
	CKO	Oar2-Ires Cre	E12.5	Down	Palate bone formation ↓	^56^
	CKO	Mx1 Cre^PolyI:C^	Early induction: P3–P7, Late induction: P21–P25	Down	Bone mass ↑, bone formation ↑	^49^
	CKO	3.2 kb Col1 Cre^ER^	E13.5–E18.5, P2–P10/P14, P2–P20/P21, 8–10/12 w, 8–22 w	Down	Bone mass ↑, bone formation ↓, bone resorption ↓, osteoblast proliferation ↑, osteoblast differentiation ↓, osteoclast number ↓	^58,60,65^
	CKO	2.3 kb Col1 Cre	P2, 5 w, 8 w	Down	Bone mass ↑, bone formation ↓, bone resorption ↓, osteoblast number ↑, osteoclast number ↓	^59^
	CKO	Og2 Cre	3 m, 10 m	Down	Bone mass (early ↓, late ↑), bone formation ↓, bone resorption ↓, osteoblast differentiation ↓	^61^
	CKO	Ctsk Cre	8 w, 12 w	Down	Bone mass ↑, bone formation ↑, bone resorption ↓, osteoblast number ↑	^59^
	CKO	Col2 Cre	E14.5	Down	Generalized chondrodysplasia	^80^
	CKO	Gdf5 Cre	1 w, 2 w, 7 w, 9 w	Down	Cartilage extracellular matrix ↓	^83^
	CKO	Aggrecan Cre^ER^	1 w, 2 w, 1 m, 2 m, 5 m	Down	Arrested endochondral bone formation, ectopic bone and fibrous formation, chondrocyte proliferation and differentiation ↓	^84,85,86^
	Tg (*caAlk3*)	Col2	E13.5, E17.5	Up	Chondrocyte maturation ↑	^82^
	CKI (UAS-*caAlk3*)	Col2 Gal4	E17.5	Up	Perinatal lethality, short long bone and growth plate	^82^
*ALK6*	KO		E12.5, E13.5, E14.5, E17.5, P0	Down	Restricted chondrodysplasia, chondrocyte proliferation and differentiation ↓	^80,82,88^
	KO		E11.5, E12.5, E13.5/14, E16.5	Down	Restricted chondrodysplasia, mesenchymal cell proliferation and differentiation ↓	^92^
	Tg (truncated *Alk6*)	2.3 kb Col1	E18.5, 1 m, 6 w, 8 w, 10 w, 12 w	Down	Bone mass ↓, bone formation ↓, bone mineral density ↓, osteoblast number ↓, osteoblast differentiation ↓	^93^

BMP, bone morphogenetic protein; m, month; w, week.
